# 3-(4-Methyl­phen­yl)-4-[(thio­semi­carba­zono)meth­yl]-1,2,3-oxa­diazol-3-ium-5-olate 1,4-dioxane hemisolvate

**DOI:** 10.1107/S1600536813008805

**Published:** 2013-04-13

**Authors:** M. Abdul Rahiman, G. N. Ravikumar, Wan-Sin Loh, Ibrahim Abdul Razak

**Affiliations:** a Department of PG Studies in Chemistry, Government Science College, Hassan 573 201, India; b X-ray Crystallography Unit, School of Physics, Universiti Sains Malaysia, 11800 USM, Penang, Malaysia

## Abstract

The asymmetric unit of the title compound, C_11_H_11_N_5_O_2_S·0.5C_4_H_8_O_2_, contains one 3-(*p*-tol­yl)sydnone 4-thio­semi­carba­zone mol­ecule and a half mol­ecule of 1,4-dioxane, which lies abount an inversion centre. The sydnone ring is almost planar, with a maximum deviation of 0.002 (1) Å, and forms a dihedral angle of 46.31 (5)° with the benzene ring. In the crystal, the two components are linked into a tape along [01-1] by N—H⋯O and N—H⋯S hydrogen bonds. The crystal structure is further stabilized by C—H⋯O and C—H⋯π inter­actions, forming a three-dimensional network.

## Related literature

For the biological acitivity of sydnones, see: Rai *et al.* (2008 ▶); Jyothi *et al.* (2008 ▶); Nithinchandra *et al.* (2012 ▶); Kalluraya *et al.* (2001 ▶). For a related structure, see: Fun *et al.* (2011 ▶). For the stability of the temperature controller used for the data collection, see: Cosier & Glazer (1986 ▶).
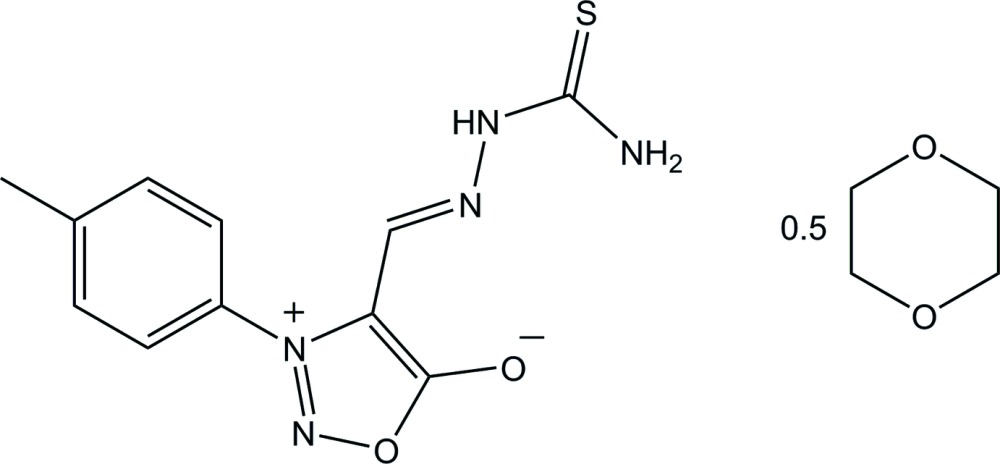


## Experimental

### 

#### Crystal data

C_11_H_11_N_5_O_2_S·0.5C_4_H_8_O_2_
*M* *_r_* = 321.36
Triclinic, 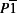

*a* = 7.7463 (1) Å
*b* = 9.3776 (1) Å
*c* = 10.4449 (2) Å
α = 79.689 (1)°
β = 87.168 (1)°
γ = 87.461 (1)°
*V* = 745.09 (2) Å^3^
*Z* = 2
Mo *K*α radiation
μ = 0.24 mm^−1^
*T* = 100 K
0.35 × 0.29 × 0.24 mm

#### Data collection

Bruker APEXII CCD area-detector diffractometer
Absorption correction: multi-scan (*SADABS*; Bruker, 2009 ▶) *T*_min_ = 0.921, *T*_max_ = 0.944
19865 measured reflections
5407 independent reflections
4782 reflections with *I* > 2σ(*I*)
*R*_int_ = 0.021

#### Refinement

*R*[*F* ^2^ > 2σ(*F* ^2^)] = 0.035
*wR*(*F* ^2^) = 0.090
*S* = 1.08
5407 reflections
212 parameters
H atoms treated by a mixture of independent and constrained refinement
Δρ_max_ = 0.43 e Å^−3^
Δρ_min_ = −0.24 e Å^−3^

### 

Data collection: *APEX2* (Bruker, 2009 ▶); cell refinement: *SAINT* (Bruker, 2009 ▶); data reduction: *SAINT*; program(s) used to solve structure: *SHELXTL* (Sheldrick, 2008 ▶); program(s) used to refine structure: *SHELXTL*; molecular graphics: *SHELXTL*; software used to prepare material for publication: *SHELXTL* and *PLATON* (Spek, 2009 ▶).

## Supplementary Material

Crystal structure: contains datablock(s) global, I. DOI: 10.1107/S1600536813008805/is5258sup1.cif

Structure factors: contains datablock(s) I. DOI: 10.1107/S1600536813008805/is5258Isup2.hkl

Supplementary material file. DOI: 10.1107/S1600536813008805/is5258Isup3.cml

Additional supplementary materials:  crystallographic information; 3D view; checkCIF report

## Figures and Tables

**Table 1:** Hydrogen-bond geometry (Å, °) *Cg*1 is the centroid of the C1–C6 benzene ring.

*D*—H⋯*A*	*D*—H	H⋯*A*	*D*⋯*A*	*D*—H⋯*A*
N4—H1*N*4⋯O3^i^	0.872 (17)	2.040 (17)	2.8624 (12)	156.8 (14)
N5—H1*N*5⋯S1^ii^	0.872 (18)	2.613 (18)	3.4754 (9)	170.2 (14)
C5—H5*A*⋯O2^iii^	0.95	2.31	3.2215 (13)	162
C9—H9*A*⋯O3^i^	0.95	2.34	3.1394 (12)	141
C12—H12*B*⋯O2^iv^	0.99	2.56	3.2626 (14)	128
C11—H11*A*⋯*Cg*1^v^	0.98	2.94	3.5736 (12)	123

Symmetry codes: (i) 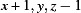; (ii) 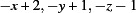; (iii) 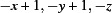; (iv) 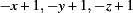; (v) 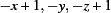.

**Fig. 1.**
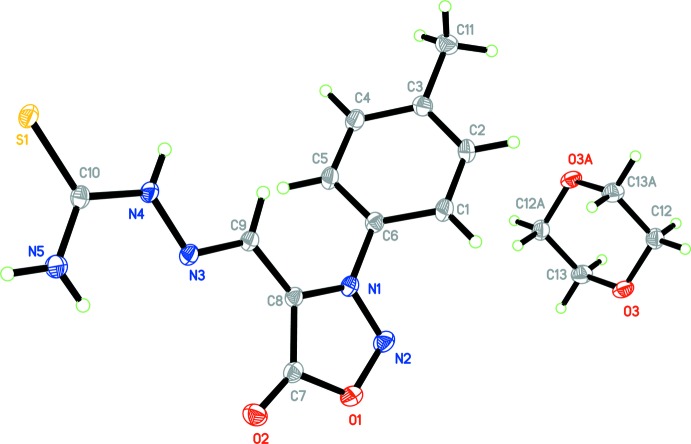
The molecular structure of the title compound, showing 50% probability displacement ellipsoids.

**Fig. 2.**
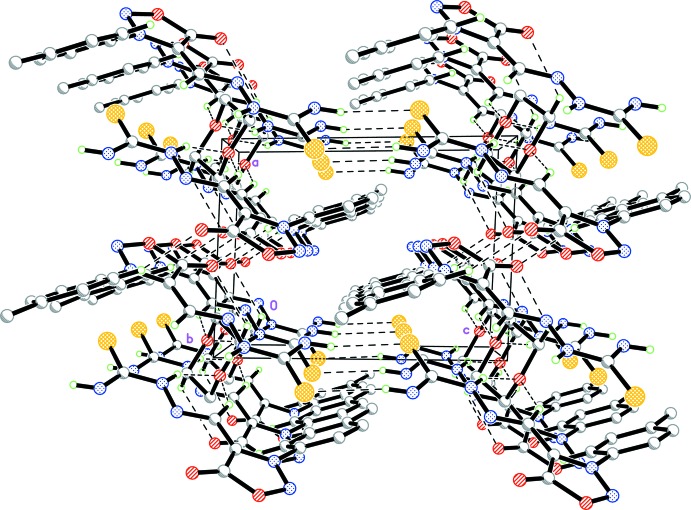
The crystal packing of the title compound, viewed along the b axis. H atoms not involved in the intermolecular interactions (dashed lines) have been omitted for clarity.

## supplementary crystallographic information

### Comment

Sydnones are mesoionic heterocyclic aromatic compounds. The study of sydnones still remains a field of interest because of their electronic structures and also because of the varied types of biological activities displayed by some of them (Rai *et al.*, 2008). Recently sydnone derivatives were found to exhibit promising antimicrobial (Jyothi *et al.*, 2008), anti-inflammatory (Nithinchandra *et al.*, 2012) and *CNS* depressant properties (Kalluraya *et al.*, 2001). Since their discovery, sydnones have shown diverse biological activities and it is thought that the *meso*-ionic nature of the sydnone ring promotes significant interactions with biological systems.

The asymmetric unit of the title compound, Fig. 1, contains one 3-(*p*-tolyl)-sydnone-4-thiosemicarbazone molecule and half of a 1,4-dioxane molecule. The sydnone ring (N1/N2/O1/C7/C8) is almost planar with maximum deviation of 0.002 (1) Å at O1 and it forms dihedral angle of 46.31 (5)° with the benzene ring (C1–C6).The complete 1–4 dioxane molecule is generated by crystallograhic inversion symmetry [symmetry code = -*x*, -*y*, -*z* + 2]. Bond lengths and angles are almost comparable with the related structure (Fun *et al.*, 2011).

In the crystal structure, Fig. 2, the molecules are linked into three dimensional network by intermolecular N4—H1N4···O3, N5—H1N5···S1, C5—H5A···O2, C9—H9A···O3 and C12—H12B···O2 hydrogen bonds (Table 1). The crystal structure was further stabilized by C—H···π interactions (Table 1), involving the centroid of the benzene ring (*Cg*1).

### Experimental

To a mixture of 4-formyl-3-(*p*-tolyl)sydnone (0.01 mol) and thiosemicarbazide (0.01 mol) in ethanol, a catalytic amount of concentrated H_2_SO_4_ was added. The solution was stirred at room temperature for 23 h. The solid product that separated out was filtered and dried. The recrystallization of the sample was done using an ethanol-dioxane (1:1 *v*/*v*) mixture. The slow evaporation of the ethanol-dioxane mixture of the compound resulted in crystals suitable for X-ray analysis.

### Refinement

N-bound H atoms were located in a difference Fourier map and were refined freely [N—H = 0.868 (17) to 0.872 (18) Å]. The remaining H atoms were located geometrically and were refined using a riding model with *U*_iso_(H) = 1.2 or 1.5*U*_eq_(C) (C—H = 0.95 to 0.99 Å). A rotating group model was applied to the methyl group. In the final refinement, two outliners (-3 6 14 and 1 8 14) were omitted.

### Crystal data

**Table:**

C_11_H_11_N_5_O_2_S·0.5C_4_H_8_O_2_	*Z* = 2
*M**_r_* = 321.36	*F*(000) = 336
Triclinic, *P*1	*D*_x_ = 1.432 Mg m^−^^3^
Hall symbol: -P 1	Mo *K*α radiation, λ = 0.71073 Å
*a* = 7.7463 (1) Å	Cell parameters from 9931 reflections
*b* = 9.3776 (1) Å	θ = 2.2–32.7°
*c* = 10.4449 (2) Å	µ = 0.24 mm^−^^1^
α = 79.689 (1)°	*T* = 100 K
β = 87.168 (1)°	Block, yellow
γ = 87.461 (1)°	0.35 × 0.29 × 0.24 mm
*V* = 745.09 (2) Å^3^	

### Data collection

**Table:**

Bruker APEXII CCD area-detector diffractometer	5407 independent reflections
Radiation source: fine-focus sealed tube	4782 reflections with *I* > 2σ(*I*)
Graphite monochromator	*R*_int_ = 0.021
φ and ω scans	θ_max_ = 32.7°, θ_min_ = 2.0°
Absorption correction: multi-scan (*SADABS*; Bruker, 2009)	*h* = −11→11
*T*_min_ = 0.921, *T*_max_ = 0.944	*k* = −14→14
19865 measured reflections	*l* = −15→15

### Refinement

**Table:**

Refinement on *F*^2^	Primary atom site location: structure-invariant direct methods
Least-squares matrix: full	Secondary atom site location: difference Fourier map
*R*[*F*^2^ > 2σ(*F*^2^)] = 0.035	Hydrogen site location: inferred from neighbouring sites
*wR*(*F*^2^) = 0.090	H atoms treated by a mixture of independent and constrained refinement
*S* = 1.08	*w* = 1/[σ^2^(*F*_o_^2^) + (0.0369*P*)^2^ + 0.310*P*] where *P* = (*F*_o_^2^ + 2*F*_c_^2^)/3
5407 reflections	(Δ/σ)_max_ = 0.002
212 parameters	Δρ_max_ = 0.43 e Å^−^^3^
0 restraints	Δρ_min_ = −0.24 e Å^−^^3^

### Special details

**Table:**

Experimental. The crystal was placed in the cold stream of an Oxford Cryosystems Cobra open-flow nitrogen cryostat (Cosier & Glazer, 1986) operating at 100.0 (1) K.
Geometry. All e.s.d.'s (except the e.s.d. in the dihedral angle between two l.s. planes) are estimated using the full covariance matrix. The cell e.s.d.'s are taken into account individually in the estimation of e.s.d.'s in distances, angles and torsion angles; correlations between e.s.d.'s in cell parameters are only used when they are defined by crystal symmetry. An approximate (isotropic) treatment of cell e.s.d.'s is used for estimating e.s.d.'s involving l.s. planes.
Refinement. Refinement of *F*^2^ against ALL reflections. The weighted *R*-factor *wR* and goodness of fit *S* are based on *F*^2^, conventional *R*-factors *R* are based on *F*, with *F* set to zero for negative *F*^2^. The threshold expression of *F*^2^ > σ(*F*^2^) is used only for calculating *R*-factors(gt) *etc*. and is not relevant to the choice of reflections for refinement. *R*-factors based on *F*^2^ are statistically about twice as large as those based on *F*, and *R*- factors based on ALL data will be even larger.

### Fractional atomic coordinates and isotropic or equivalent isotropic displacement parameters (Å^2^)

**Table:**

	*x*	*y*	*z*	*U*_iso_*/*U*_eq_	
S1	1.09153 (3)	0.32862 (3)	−0.33706 (2)	0.01659 (6)	
O1	0.47671 (10)	0.67385 (8)	0.20795 (7)	0.01898 (15)	
O2	0.57636 (11)	0.75787 (8)	0.00058 (8)	0.02035 (15)	
N1	0.59090 (11)	0.46077 (9)	0.24338 (8)	0.01427 (15)	
N2	0.48951 (12)	0.54926 (10)	0.29962 (9)	0.01841 (17)	
N3	0.79973 (11)	0.49150 (9)	−0.07807 (8)	0.01443 (15)	
N4	0.91376 (11)	0.40423 (9)	−0.13781 (8)	0.01460 (15)	
N5	0.90336 (13)	0.57238 (10)	−0.32586 (9)	0.01962 (17)	
C1	0.68059 (14)	0.31717 (11)	0.44782 (10)	0.01739 (18)	
H1A	0.6855	0.4040	0.4823	0.021*	
C2	0.72069 (14)	0.18334 (11)	0.52322 (10)	0.01834 (18)	
H2A	0.7512	0.1787	0.6109	0.022*	
C3	0.71703 (13)	0.05535 (11)	0.47262 (10)	0.01713 (18)	
C4	0.66732 (14)	0.06363 (11)	0.34462 (10)	0.01855 (18)	
H4A	0.6633	−0.0228	0.3095	0.022*	
C5	0.62369 (14)	0.19592 (11)	0.26773 (10)	0.01751 (18)	
H5A	0.5883	0.2008	0.1812	0.021*	
C6	0.63314 (13)	0.32100 (10)	0.32082 (9)	0.01490 (17)	
C7	0.57264 (13)	0.65882 (11)	0.09278 (10)	0.01562 (17)	
C8	0.64729 (12)	0.51563 (10)	0.11978 (9)	0.01360 (16)	
C9	0.76555 (12)	0.44016 (10)	0.04353 (9)	0.01363 (16)	
H9A	0.8197	0.3512	0.0827	0.016*	
C10	0.96088 (12)	0.44310 (10)	−0.26539 (9)	0.01415 (16)	
C11	0.76728 (15)	−0.08866 (12)	0.55341 (11)	0.0222 (2)	
H11A	0.6967	−0.1643	0.5313	0.033*	
H11B	0.7480	−0.0838	0.6460	0.033*	
H11C	0.8898	−0.1117	0.5356	0.033*	
O3	−0.07175 (11)	0.12953 (8)	1.03347 (8)	0.02138 (16)	
C12	0.07792 (15)	0.05932 (12)	1.09562 (11)	0.0217 (2)	
H12A	0.0419	−0.0077	1.1759	0.026*	
H12B	0.1517	0.1328	1.1203	0.026*	
C13	−0.17918 (15)	0.02410 (13)	0.99557 (12)	0.0237 (2)	
H13A	−0.2815	0.0737	0.9518	0.028*	
H13B	−0.2203	−0.0436	1.0739	0.028*	
H1N4	0.947 (2)	0.3189 (18)	−0.0967 (16)	0.028 (4)*	
H1N5	0.919 (2)	0.5963 (19)	−0.4101 (18)	0.036 (4)*	
H2N5	0.826 (2)	0.6209 (18)	−0.2864 (16)	0.030 (4)*	

### Atomic displacement parameters (Å^2^)

**Table:**

	*U*^11^	*U*^22^	*U*^33^	*U*^12^	*U*^13^	*U*^23^
S1	0.02010 (12)	0.01732 (11)	0.01242 (10)	0.00041 (8)	0.00249 (8)	−0.00394 (8)
O1	0.0199 (3)	0.0181 (3)	0.0193 (3)	0.0032 (3)	0.0007 (3)	−0.0054 (3)
O2	0.0241 (4)	0.0157 (3)	0.0206 (4)	0.0000 (3)	−0.0040 (3)	−0.0009 (3)
N1	0.0148 (3)	0.0160 (3)	0.0123 (3)	−0.0012 (3)	0.0019 (3)	−0.0038 (3)
N2	0.0190 (4)	0.0195 (4)	0.0168 (4)	0.0016 (3)	0.0036 (3)	−0.0054 (3)
N3	0.0155 (3)	0.0152 (3)	0.0129 (3)	−0.0005 (3)	0.0013 (3)	−0.0036 (3)
N4	0.0182 (4)	0.0145 (3)	0.0107 (3)	0.0008 (3)	0.0022 (3)	−0.0021 (3)
N5	0.0247 (4)	0.0181 (4)	0.0139 (4)	0.0032 (3)	0.0046 (3)	0.0006 (3)
C1	0.0214 (4)	0.0179 (4)	0.0133 (4)	−0.0031 (4)	0.0020 (3)	−0.0042 (3)
C2	0.0215 (5)	0.0214 (4)	0.0123 (4)	−0.0021 (4)	−0.0009 (3)	−0.0031 (3)
C3	0.0170 (4)	0.0184 (4)	0.0149 (4)	−0.0002 (3)	0.0018 (3)	−0.0007 (3)
C4	0.0240 (5)	0.0168 (4)	0.0153 (4)	−0.0014 (4)	0.0013 (3)	−0.0042 (3)
C5	0.0224 (5)	0.0180 (4)	0.0125 (4)	−0.0027 (4)	0.0012 (3)	−0.0037 (3)
C6	0.0166 (4)	0.0155 (4)	0.0121 (4)	−0.0018 (3)	0.0025 (3)	−0.0015 (3)
C7	0.0158 (4)	0.0161 (4)	0.0160 (4)	−0.0012 (3)	−0.0012 (3)	−0.0056 (3)
C8	0.0149 (4)	0.0138 (4)	0.0120 (4)	−0.0007 (3)	0.0013 (3)	−0.0026 (3)
C9	0.0148 (4)	0.0139 (4)	0.0124 (4)	−0.0012 (3)	0.0007 (3)	−0.0032 (3)
C10	0.0149 (4)	0.0155 (4)	0.0122 (4)	−0.0027 (3)	0.0012 (3)	−0.0026 (3)
C11	0.0242 (5)	0.0199 (5)	0.0206 (5)	0.0029 (4)	−0.0003 (4)	0.0004 (4)
O3	0.0263 (4)	0.0143 (3)	0.0233 (4)	0.0020 (3)	0.0021 (3)	−0.0041 (3)
C12	0.0271 (5)	0.0205 (5)	0.0178 (5)	−0.0019 (4)	−0.0015 (4)	−0.0036 (4)
C13	0.0207 (5)	0.0227 (5)	0.0281 (5)	0.0016 (4)	−0.0007 (4)	−0.0060 (4)

### Geometric parameters (Å, º)

**Table:**

S1—C10	1.6893 (10)	C3—C11	1.5051 (14)
O1—N2	1.3749 (12)	C4—C5	1.3897 (14)
O1—C7	1.4079 (12)	C4—H4A	0.9500
O2—C7	1.2126 (12)	C5—C6	1.3903 (14)
N1—N2	1.3122 (11)	C5—H5A	0.9500
N1—C8	1.3597 (12)	C7—C8	1.4244 (13)
N1—C6	1.4449 (13)	C8—C9	1.4306 (13)
N3—C9	1.2939 (12)	C9—H9A	0.9500
N3—N4	1.3797 (11)	C11—H11A	0.9800
N4—C10	1.3534 (12)	C11—H11B	0.9800
N4—H1N4	0.871 (16)	C11—H11C	0.9800
N5—C10	1.3316 (13)	O3—C12	1.4329 (14)
N5—H1N5	0.872 (18)	O3—C13	1.4395 (14)
N5—H2N5	0.868 (17)	C12—C13^i^	1.5076 (16)
C1—C6	1.3878 (14)	C12—H12A	0.9900
C1—C2	1.3884 (14)	C12—H12B	0.9900
C1—H1A	0.9500	C13—C12^i^	1.5076 (16)
C2—C3	1.3972 (14)	C13—H13A	0.9900
C2—H2A	0.9500	C13—H13B	0.9900
C3—C4	1.3974 (14)		
			
N2—O1—C7	111.07 (7)	O2—C7—C8	135.35 (10)
N2—N1—C8	114.90 (8)	O1—C7—C8	104.14 (8)
N2—N1—C6	116.69 (8)	N1—C8—C7	105.26 (8)
C8—N1—C6	128.37 (8)	N1—C8—C9	123.94 (9)
N1—N2—O1	104.63 (7)	C7—C8—C9	130.70 (9)
C9—N3—N4	113.44 (8)	N3—C9—C8	120.84 (9)
C10—N4—N3	120.28 (8)	N3—C9—H9A	119.6
C10—N4—H1N4	118.9 (11)	C8—C9—H9A	119.6
N3—N4—H1N4	120.4 (11)	N5—C10—N4	117.10 (9)
C10—N5—H1N5	119.6 (12)	N5—C10—S1	124.08 (7)
C10—N5—H2N5	119.2 (11)	N4—C10—S1	118.81 (7)
H1N5—N5—H2N5	118.8 (15)	C3—C11—H11A	109.5
C6—C1—C2	118.34 (9)	C3—C11—H11B	109.5
C6—C1—H1A	120.8	H11A—C11—H11B	109.5
C2—C1—H1A	120.8	C3—C11—H11C	109.5
C1—C2—C3	121.26 (9)	H11A—C11—H11C	109.5
C1—C2—H2A	119.4	H11B—C11—H11C	109.5
C3—C2—H2A	119.4	C12—O3—C13	110.28 (8)
C2—C3—C4	118.66 (9)	O3—C12—C13^i^	109.89 (9)
C2—C3—C11	120.90 (9)	O3—C12—H12A	109.7
C4—C3—C11	120.44 (9)	C13^i^—C12—H12A	109.7
C5—C4—C3	121.25 (9)	O3—C12—H12B	109.7
C5—C4—H4A	119.4	C13^i^—C12—H12B	109.7
C3—C4—H4A	119.4	H12A—C12—H12B	108.2
C4—C5—C6	118.22 (9)	O3—C13—C12^i^	109.94 (9)
C4—C5—H5A	120.9	O3—C13—H13A	109.7
C6—C5—H5A	120.9	C12^i^—C13—H13A	109.7
C1—C6—C5	122.23 (9)	O3—C13—H13B	109.7
C1—C6—N1	117.89 (9)	C12^i^—C13—H13B	109.7
C5—C6—N1	119.87 (9)	H13A—C13—H13B	108.2
O2—C7—O1	120.50 (9)		
			
C8—N1—N2—O1	−0.38 (11)	N2—O1—C7—O2	179.80 (9)
C6—N1—N2—O1	177.55 (8)	N2—O1—C7—C8	−0.36 (10)
C7—O1—N2—N1	0.46 (10)	N2—N1—C8—C7	0.17 (11)
C9—N3—N4—C10	179.48 (9)	C6—N1—C8—C7	−177.48 (9)
C6—C1—C2—C3	1.24 (16)	N2—N1—C8—C9	176.73 (9)
C1—C2—C3—C4	−1.73 (16)	C6—N1—C8—C9	−0.92 (16)
C1—C2—C3—C11	177.64 (10)	O2—C7—C8—N1	179.92 (12)
C2—C3—C4—C5	0.61 (16)	O1—C7—C8—N1	0.12 (10)
C11—C3—C4—C5	−178.75 (10)	O2—C7—C8—C9	3.7 (2)
C3—C4—C5—C6	0.93 (16)	O1—C7—C8—C9	−176.11 (10)
C2—C1—C6—C5	0.39 (15)	N4—N3—C9—C8	−178.91 (8)
C2—C1—C6—N1	179.72 (9)	N1—C8—C9—N3	172.48 (9)
C4—C5—C6—C1	−1.46 (15)	C7—C8—C9—N3	−11.90 (16)
C4—C5—C6—N1	179.22 (9)	N3—N4—C10—N5	5.06 (14)
N2—N1—C6—C1	−45.36 (13)	N3—N4—C10—S1	−176.46 (7)
C8—N1—C6—C1	132.25 (11)	C13—O3—C12—C13^i^	58.64 (12)
N2—N1—C6—C5	133.99 (10)	C12—O3—C13—C12^i^	−58.67 (12)
C8—N1—C6—C5	−48.40 (14)		

Symmetry code: (i) −*x*, −*y*, −*z*+2.

### Hydrogen-bond geometry (Å, º)

Cg1 is the centroid of the C1–C6 benzene ring.

**Table:**

*D*—H···*A*	*D*—H	H···*A*	*D*···*A*	*D*—H···*A*
N4—H1*N*4···O3^ii^	0.872 (17)	2.040 (17)	2.8624 (12)	156.8 (14)
N5—H1*N*5···S1^iii^	0.872 (18)	2.613 (18)	3.4754 (9)	170.2 (14)
C5—H5*A*···O2^iv^	0.95	2.31	3.2215 (13)	162
C9—H9*A*···O3^ii^	0.95	2.34	3.1394 (12)	141
C12—H12*B*···O2^v^	0.99	2.56	3.2626 (14)	128
C11—H11*A*···*Cg*1^vi^	0.98	2.94	3.5736 (12)	123

Symmetry codes: (ii) *x*+1, *y*, *z*−1; (iii) −*x*+2, −*y*+1, −*z*−1; (iv) −*x*+1, −*y*+1, −*z*; (v) −*x*+1, −*y*+1, −*z*+1; (vi) −*x*+1, −*y*, −*z*+1.
